# Multimodality imaging in a young patient with ST-Elevation Myocardial Infarction

**DOI:** 10.1093/ehjcr/ytag666

**Published:** 2026-09-04

**Authors:** Edwin Adhi Darmawan Batubara, Celly Anantaria Atmadikoesoemah, Vidya Gilang Rejeki, Bambang Widyantoro

**Affiliations:** Department of Cardiology and Vascular Medicine, Faculty of Medicine, Universitas Indonesia, National Cardiovascular Center Harapan Kita, 11420 Jakarta, Indonesia; Department of Cardiology and Vascular Medicine, Faculty of Medicine, Universitas Indonesia, National Cardiovascular Center Harapan Kita, 11420 Jakarta, Indonesia; Department of Cardiology and Vascular Medicine, Faculty of Medicine, Universitas Indonesia, National Cardiovascular Center Harapan Kita, 11420 Jakarta, Indonesia; Department of Cardiology and Vascular Medicine, Faculty of Medicine, Universitas Indonesia, National Cardiovascular Center Harapan Kita, 11420 Jakarta, Indonesia

## Case description

**Figure 1:**
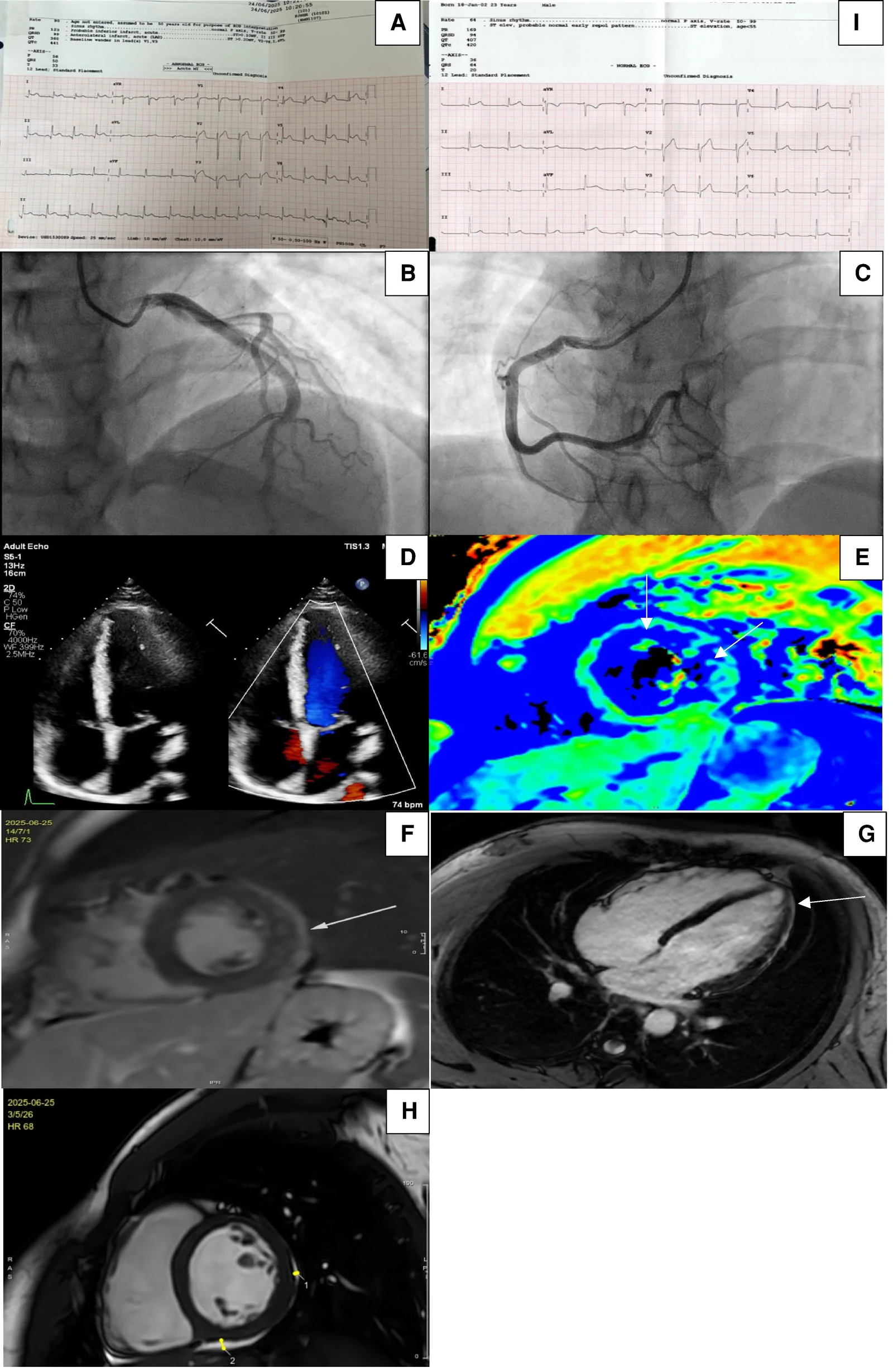
(*A*) Electrocardiogram on admission with ST elevation in the anterolateral leads. (*B* and *C*) Coronary angiography, (*B*) normal left coronary artery, (*C*) normal right coronary artery. (*D*) Transthoracic echocardiogram showed no regional wall motion abnormality with good ejection fraction and no pericardial effusion. (*E–G*) Cardiac magnetic resonance imaging, (*E*) increased T2 relaxation time in mid-anterior and mid-lateral (white arrow). (*F* and *G*) Subepicardial and pericardial late gadolinium enhancement in apicolateral (white arrow). (*H*) Pericardial effusion. (*I*) Serial electrocardiogram evaluation showed normalization of the ST-segment before discharge.

A 23-year-old male came to the emergency department with substernal chest pain 5 h before admission. He also experienced nausea and cold sweats. The patient had a history of flu-like symptoms with fever 3 days ago. He denied any history of hypertension, dyslipidaemia, or smoking. Electrocardiogram examination showed anterolateral leads ST elevation (*Figure 1A*). High-sensitivity Troponin T (Hs Trop T) examination revealed a level of 530 ng/L, and C-reactive protein was increased to 43 mg/L. The patient was sent to the catheterization laboratory, and angiography showed normal coronary arteries (*Figure 1B* and *C*). Transthoracic echocardiography revealed a normal ejection fraction, and no pericardial effusion was seen (*Figure 1D*).

Later, the patient underwent cardiac magnetic resonance imaging and showed subtle subepicardial and epicardial late gadolinium enhancement in the apical lateral region, mild circumferential pericardial effusion (1.5–4.5 mm), and increased T2 relaxation time in mid-anterior and mid-lateral, confirming the diagnosis of perimyocarditis (*Figure 1E–H*). The patient was treated with ibuprofen and colchicine. The patient was discharged on Day 5 with resolved chest pain discomfort, with serial Hs Trop T 12 ng/L and serial ECG examination showing normalization of the ST-segment (*Figure 1I*).

Acute ST-segment elevation condition in the young population remains challenging to diagnose and potentially leads to delayed treatment. Acute perimyocarditis should always be considered in this condition.^1,2^ This case highlights the use of multimodality imaging in the diagnosis of perimyocarditis. Cardiac magnetic resonance has played an essential role in enabling the identification and characterization of tissue.^1,3^ Accurate diagnosis is critical for appropriate therapy and preventing further complications.

## Lead author biography


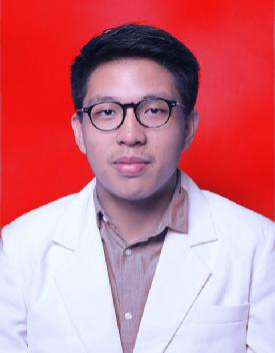


Edwin Adhi D. Batubara is a medical doctor who graduated from the University of Indonesia. He is currently resident in Department of Cardiology and Vascular Medicine, Faculty of Medicine, Universitas Indonesia, National Cardiovascular Center Harapan Kita, Jakarta, Indonesia

## Data Availability

All relevant data are presented within the manuscript.
